# Arterial Tortuosity Syndrome: Unraveling a Rare Vascular Disorder

**DOI:** 10.7759/cureus.44906

**Published:** 2023-09-08

**Authors:** Chukwuyem Ekhator, Monika Devi, Chad Barker, Shamayel Safdar, Rabbia Irfan, Jahnavi Malineni, Iqbal Hussain, Pakeezah Bisharat, Afif Ramadhan, Ali M Abdelaziz, Sophia B Bellegarde, Muhammad Nabeel Saddique

**Affiliations:** 1 Neuro-Oncology, College of Osteopathic Medicine, New York Institute of Technology, Old Westbury, USA; 2 Medicine, Ziauddin University, Karachi, PAK; 3 Public Health, University of South Florida, Tampa, USA; 4 Internal Medicine, Mayo Hospital, Lahore, PAK; 5 Medicine and Surgery, Maharajah's Institute of Medical Sciences, Vizianagaram, IND; 6 Medicine and Surgery, Khyber Medical University, Peshawar, PAK; 7 Internal Medicine, Khyber Medical University, Peshawar, PAK; 8 Medicine, Universal Scientific Education and Research Network (USERN), Yogyakarta, IDN; 9 Medicine, Faculty of Medicine, Public Health, and Nursing, Gadjah Mada University, Yogyakarta, IDN; 10 Internal Medicine, Alexandria University Faculty of Medicine, Alexandria, EGY; 11 Pathology and Laboratory Medicine, American University of Antigua, St. John's, ATG; 12 Medicine and Surgery, Mayo Hospital, Lahore, PAK

**Keywords:** vascular pathology, inherited arteriopathy, loeys-dietz syndrome, marfan syndrome, arterial tortuosity syndrome

## Abstract

Arterial tortuosity syndrome (ATS) is a rare genetic disorder characterized by abnormal twists and turns of arteries, leading to cardiovascular complications. This syndrome, first reported around 55 years ago, is inherited in an autosomal recessive manner and affects both genders. ATS manifests primarily in childhood, with arterial abnormalities disrupting blood circulation, increasing shear stress, and causing complications, such as atherosclerosis and strokes. This article reviews the genetics, etiology, pathophysiology, clinical presentation, diagnosis, associated conditions, management, and challenges of ATS. The syndrome's genetic cause is linked to mutations in the SLC2A10 gene, affecting collagen and elastin synthesis. Arterial tortuosity, a complex phenomenon, arises from factors such as vessel elongation, anatomic fixation, and vessel diameter. ATS is one of many conditions associated with arterial tortuosity, including Marfan syndrome and Loeys-Dietz syndrome. Recent studies highlight arterial tortuosity's potential as a prognostic indicator for adverse cardiovascular events. Management requires a multidisciplinary approach, and surveillance and prevention play key roles. Despite challenges, advancements in understanding ATS offer hope for targeted therapies and improved patient care.

## Introduction and background

Arterial tortuosity syndrome (ATS) is a rare connective tissue disorder characterized by abnormal twists and turns (tortuosity) of the arteries. The condition was first reported around 55 years ago and is inherited in an autosomal recessive manner [1,2]. It affects both males and females, with no significant gender bias [3]. ATS usually manifests in early childhood, although cases have been diagnosed in adults as well as during prenatal stages [4,5]. The tortuosity of arteries disrupts blood circulation, increases vessel wall shear stress, and can lead to complications such as atherosclerosis and cerebrovascular strokes. Chronic hypertension and vascular stiffness are also common outcomes. Patients with ATS exhibit a wide range of clinical features, including severe ventricular hypertrophy, valvular regurgitation, and occasionally atrial fibrillation. Vascular issues, such as aneurysms, dissections, and stenoses of the arteries are observed, and dysmorphic skeletal features, hyperextensible skin, hypermobile joints, and congenital contractures are distinctive physical manifestations [3,4,6]. The genetic cause of ATS has been linked to missense mutations in the SLC2A10 gene, which encodes the GLUT10 protein [3,7]. This protein is essential for the transport of ascorbate, a cofactor in collagen and elastin biosynthesis. The mutations disrupt collagen and elastin synthesis, weakening the arteries and leading to upregulated transforming growth factor-β (TGFβ) signaling [8].

Arterial tortuosity, characterized by the twisting and turning of arteries, has been recognized for many years [9]. While it has historically been associated with factors such as aging, female sex, and high blood pressure, contemporary research has identified it as a hallmark of genetic arteriopathies [10]. ATS is one such condition in which arterial tortuosity is a key feature. It is important to standardize the definition, measurement, and criteria for normalcy of arterial tortuosity for its incorporation into clinical practice. Tortuosity can occur in arteries of various sizes and locations, from small vessels such as capillaries to larger ones, like the aorta. There are different terms used to describe the types of tortuosity, including S-shaped curves, looping, and coiling [11]. Mechanically, arterial tortuosity is associated with factors such as blood pressure, blood flow, axial tension, and structural changes in the vessel walls. Anatomic factors such as vessel fixation points and branch points play a role in the development of tortuosity [12].

ATS is not the only condition associated with arterial tortuosity. Several genetically mediated conditions, including Loeys-Dietz syndrome (LDS), Marfan syndrome, and others, exhibit tortuosity as a feature. For instance, LDS due to mutations in the TGFBR1 and TGFBR2 genes is characterized by diffuse arterial tortuosity, particularly in the neurovascular system [13]. Marfan syndrome also involves arterial tortuosity, often observed in the vertebral and carotid arteries [14]. Turner syndrome, an X-chromosome disorder, has been associated with mild vertebral artery tortuosity [15,16]. Aneurysm-osteoarthritis syndrome caused by SMAD3 mutations exhibits arterial tortuosity, as well as Menkes disease and occipital horn syndrome. The latter conditions, caused by ATP7A mutations, are linked to arterial aneurysms along with cerebrovascular tortuosity [17,18].

Recent studies have explored the relationship between arterial tortuosity and outcomes. A study by Franken et al. on Marfan syndrome patients indicated that higher arterial tortuosity was associated with an increased risk of aortic dissection and adverse cardiovascular events [19]. Other studies have suggested that altered flow profiles through tortuous arteries could lead to more severe distal aortic phenotypes and susceptibility to aortic dissections [10]. In conclusion, ATS is a complex phenomenon influenced by mechanical and anatomic factors, and it is a feature observed in various genetically mediated conditions. Recent research highlights the importance of arterial tortuosity as a potential prognostic indicator for adverse cardiovascular events in certain conditions. Understanding the genetics and etiology of ATS, as well as its broader implications in vascular biology, can contribute to improved diagnosis, management, and treatment strategies for this condition.

## Review

Pathophysiology

ATS is a rare genetic disorder characterized by abnormal elongation, tortuosity, and sometimes stenosis or aneurysm formation in major arteries. The pathophysiology of ATS is complex and involves various anatomical, mechanical, and genetic factors. Clinical and experimental studies have shown that mechanical factors, such as blood pressure, blood flow, axial tension, and wall structural changes, play a significant role in the development of arterial tortuosity. Here, we delve into the pathophysiological mechanisms underlying ATS based on the provided information and additional relevant sources.

Arterial tortuosity is thought to arise from abnormalities in relative vascular elongation. Several factors contribute to the development of arterial tortuosity: (1) Vascular elongation: Abnormal elongation of blood vessels can occur during primary arteriogenesis or due to factors such as growth during childhood and adolescence, further elongation in adulthood, or skeletal changes during aging. The elongation leads to redundancy in vessel length within a fixed space, causing the vessels to curve and bend [20]. (2) Anatomic fixation: Arterial elongation between fixed points contributes to tortuosity. For example, the cervical and vertebral vessels are fixed cranially by the skull and caudally by the aorta. Redundancy in length has limited space to dissipate [21]. (3) Vessel branch points: Branch points act as points of fixation, determining the pattern of tortuosity. Redundancy in vessels with multiple branch points can lead to small loops, while larger vessels may form single loops or simple S shapes [20]. (4) Vessel diameter: Vessel diameter affects the rate of change of angle per unit length. Smaller vessels can generate tighter angles over shorter distances, while larger vessels form high-amplitude, low-frequency tortuosity. (5) Dynamic tortuosity: Tortuosity may be dynamic, changing during different phases of the cardiac cycle or body position [22]. (6) Measurement considerations: When measuring tortuosity, it is crucial to consider the conditions of measurement, including factors such as vessel diameter, branch points, and the nature of the tortuosity [20].

Clinical presentation and diagnosis

ATS is a connective tissue disorder identified by a blend of cardiovascular irregularities and broad connective tissue traits. The condition is attributed to mutations in the SLC2A10 gene, pivotal in the establishment and preservation of vascular integrity. ATS is marked by extensive elongation and twisting of the aorta and mid-sized arteries, as well as localized narrowing in sections of the pulmonary arteries and/or aorta [8]. Additionally, joint hypermobility, highly stretchable skin, and various skeletal and connective tissue anomalies are prevalent characteristics of ATS [3,6,23].

The clinical presentation of ATS exhibits significant variability, ranging from severe instances with early mortality during infancy to less severe expressions during adulthood [24]. Typically emerging in early childhood, common initial indications include cardiac murmurs or cyanosis. The signs of connective tissue involvement become more pronounced with age, prompting the need for further assessment. In certain cases, infants might require admission to neonatal intensive care units due to complications such as respiratory distress syndrome. The cardiovascular system serves as the primary source of morbidity and mortality in ATS. Prominent features encompass aortic tortuosity, tortuosity observed in other arteries, aortic root aneurysm, pulmonary artery stenosis, aortic stenosis, and autonomic dysfunction. Aortic dissections, though predominantly seen in clinically diagnosed instances, can arise, and there is an elevated risk of ischemic vascular events affecting cerebrovascular circulation and abdominal arteries, potentially leading to stroke and infarctions in abdominal organs [3,8]. The management of these cardiovascular complications often necessitates surgical interventions.

Typical facial characteristics associated with ATS include a long face, down-slanted palpebral fissures, convex nasal ridge, full cheeks, micrognathia, high palate, and, in some cases, cleft palate or bifid uvula [3]. Generalized connective tissue features include joint hypermobility, joint pain, cutis laxa (stretchable skin), inguinal hernia, diaphragmatic/sliding hernia, pectus deformity, arachnodactyly, scoliosis, and other skeletal abnormalities. These features become more prominent with age [3,6].

Imaging plays a crucial role in diagnosing and assessing the extent of vascular abnormalities in ATS. Radiological signs include skin laxity observed as excessive skin folding on radiographs, skeletal deformities like kyphoscoliosis and chest wall deformities, and vascular tortuosity of the aorta and peripheral arteries [25]. The aortic elongation sign and the meandering vessel sign are characteristic findings [26]. Pulmonary artery stenosis with an inverted V configuration of the pulmonary bifurcation can also be seen [25]. The cluster of vessels sign, seen as multiple rounded vascular structures in the superior mediastinum, is another imaging observation. Multidetector CT evaluation appears to be the best choice in the investigation of ATS patients [25]. Molecular genetic testing is essential for the definitive diagnosis of ATS [6]. Mutations in the SLC2A10 gene, which codes for the GLUT10 protein, are responsible for the disorder. There are various types of mutations identified, including missense, nonsense, compound heterozygous variants, deletion variants, and intronic splice variants. Homozygous or compound heterozygous mutations in SLC2A10 result in ATS, while heterozygous carriers typically do not show vascular anomalies. The disease spectrum is wide, and the prognosis varies from severe cases with early mortality to milder manifestations [6].

Several quantitative methods have been developed to measure arterial tortuosity. These include (1) tortuosity index (TI): the ratio between the length along the centerline and the linear distance between the two endpoints, and TI accounts for the vessel's overall curvature [27]; (2) sum of angles method (SOAM): calculates the ratio between the sum of all angles at points of angulation and the length along the centerline and is effective for high-frequency, low-amplitude coils [28]; (3) inflection count metric (ICM): counts the number of inflection points along a curve, normalized by path length, and incorporates both TI and SOAM, providing a comprehensive assessment [29]; and (4) complex methods: incorporate factors like curvature and tortuosity curves to create 3D assessments [28].

Contributing factors and associated conditions

The relationship between age and arterial tortuosity has been a subject of debate and exploration. Various studies have yielded conflicting findings. In a study by Adriaans et al. involving a large patient cohort, the length of the thoracic aorta was significantly related to age, with a notable increase over the lifespan [30]. The proximal descending aorta exhibited a pronounced increase in tortuosity with age, emphasizing the role of age-related changes in vascular architecture. Furthermore, the aortic branches exhibited increased tortuosity with advancing age. These findings collectively suggest that age-related alterations in vessel structure contribute to arterial tortuosity, particularly in the thoracic aorta and its branches.

Gender has been identified as a potential factor influencing arterial tortuosity. Female sex has been associated with a higher prevalence of arterial tortuosity, especially in conditions involving smaller artery diameters. Hormonal factors, such as estrogen and progesterone, play a role in vascular remodeling and inflammation. The dynamic interplay of these hormones over a woman's lifetime affects arterial compliance, stiffness, and pulse pressure [31]. Notably, studies have demonstrated a higher prevalence of arterial tortuosity in females across various vascular beds, including the internal carotid artery and coronary arteries. The mechanisms underlying this association warrant further exploration, considering the intricate interplay between hormonal fluctuations, vascular structure, and inflammation [32].

Hypertension, a well-established risk factor for cardiovascular diseases, has been linked to arterial tortuosity. Studies have shown that people with hypertension often have vascular anomalies such as tortuosity. Animal studies have shown that hypertensive animals had a greater frequency of vascular anomalies than normotensive controls [33]. Clinical research has supported these results, demonstrating links between arterial tortuosity and arterial hypertension. Patients with hypertension have been shown to have carotid arteries and coronary arteries that are tortuous [34]. Additionally, the relationship between arterial tortuosity and hypertension includes the possibility of lacunar infarction, highlighting the potential clinical significance of these findings [35].

Diabetes mellitus, a complex metabolic disorder, has been linked to the tortuosity of small vessels, primarily arterioles [20]. While the evidence is limited, studies suggest an association between diabetes and vascular tortuosity, particularly in smaller vessels. This suggests that the microvascular alterations associated with diabetes extend to arterial tortuosity, albeit with a focus on smaller-caliber vessels.

Obesity, a growing global health concern, has also been considered a potential contributor to arterial tortuosity. Studies have demonstrated associations between body mass index (BMI) and arterial tortuosity [36,37]. Notably, higher BMI has been linked to increased carotid artery tortuosity, even after adjusting for traditional cardiovascular risk factors. The implications of this association underline the multifaceted relationship between obesity, vascular structure, and potential mechanical factors influencing arterial curvature.

Atherosclerosis, a hallmark of cardiovascular diseases, has a controversial role in the development of arterial tortuosity. While atherosclerosis itself involves vessel remodeling and structural changes, its direct link to arterial tortuosity remains less clear. Studies have explored the potential relationship between atherosclerosis and tortuosity, highlighting the complexity of their interactions [38,39]. Further research is needed to elucidate the specific mechanisms that underlie this intricate relationship.

Inherited arteriopathies encompass a diverse group of genetic syndromes that can manifest as arterial tortuosity. Syndromes such as Marfan syndrome, LDS, and aneurysms osteoarthritis syndrome exhibit varying degrees of arterial tortuosity, often in conjunction with other clinical features. The genetic basis of these syndromes is complex, involving mutations in different genes associated with connective tissue and vascular development. Understanding these inherited arteriopathies not only sheds light on the pathophysiology of arterial tortuosity but also provides valuable insights into the diagnosis and management of these conditions.

Arterial tortuosity has gained attention as a potential angiographic finding in patients with spontaneous coronary artery dissection (SCAD) [40]. Research has demonstrated a higher prevalence of coronary artery tortuosity in individuals with SCAD compared to controls [41]. Moreover, fibromuscular dysplasia (FMD) has been associated with increased coronary tortuosity, highlighting the potential interplay between these two vascular abnormalities [42]. However, a comprehensive understanding of the link between arterial tortuosity, SCAD, and FMD requires further investigation.

Management

ATS is a rare genetic disorder characterized by abnormal curvatures and twisting of the arteries throughout the body. Managing this complex condition requires a multidisciplinary approach involving various specialists to address its diverse manifestations. Although no specific clinical practice guidelines for ATS have been published, management strategies aim to address the specific symptoms and risks associated with the condition. A coordinated effort from a team of clinical geneticists, cardiologists, ophthalmologists, orthopedists, and other specialists is essential for providing comprehensive care.

After an individual is diagnosed with ATS, a series of evaluations is recommended to assess the extent of the disease and address specific concerns [6]. These evaluations include the following. (1) Aortic root aneurysm: echocardiography is recommended to measure the aortic root and interpret the measurements based on normal values for age and body size. This assessment is crucial for monitoring potential aneurysm development. (2) Arterial tortuosity (AT): A magnetic resonance angiography (MRA) or computed tomography (CT) scan with 3D reconstruction from head to pelvis helps evaluate the extent of arterial tortuosity, identify abnormal implantation of aortic branches, and detect any aneurysms or stenosis throughout the arterial tree. (3) Hypertension: Regular blood pressure measurements are essential to monitor and manage hypertension, which is a common concern in ATS. (4) Orthostatic hypotension: Tilt testing should be considered if the individual's history suggests orthostatic hypotension. (5) Palate abnormality: Evaluation for cleft palate or bifid uvula is recommended. (6) Diaphragmatic hernia/sliding hernia: Thoracoabdominal radiography helps assess the presence of these hernias. (7) Scoliosis: Clinical evaluation and skeletal radiographs assist in managing scoliosis, which is a common musculoskeletal concern in ATS. (8) Osteopenia: Bone densitometry might be considered if osteopenia is diagnosed in adulthood. (9) Ocular abnormality: Individuals should be evaluated for conditions such as keratoconus, keratoglobus, corneal thinning, and refractive errors by an ophthalmologist with expertise in connective tissue disorders. (10) Urogenital abnormalities: Ultrasound of the urinary tract can help identify urogenital abnormalities. (11) Genetic counseling: Genetic counseling by professionals experienced in genetics can provide information about the nature, mode of inheritance, and implications of ATS to aid in medical and personal decision-making.

The treatment of ATS involves managing specific manifestations and complications associated with the condition [3,6]. A comprehensive approach is necessary, considering the unique challenges posed by ATS. Some of the key treatment considerations are mentioned below (Table 1).

**Table 1:** Management of various systemic manifestations of arterial tortuosity syndrome (ATS) ATS: arterial tortuosity syndrome

Treatment of ATS Manifestations	
Aortic Aneurysm	Medical treatment with beta-adrenergic blockers or other medications like angiotensin-converting enzyme inhibitors (ACE-I) and angiotensin II receptor 1 (ATIIR1) antagonists such as losartan may be considered. However, the efficacy of these treatments in ATS has not been established. Caution is necessary when using blood pressure-lowering medications in the presence of arterial stenosis, as they may increase the risk of renal failure. Surgical treatment involving a valve-sparing procedure can be an option to prevent the need for chronic anticoagulation.
Focal Stenosis of Aorta and Aortic Branches	Catheterization and/or surgery might be required to address focal stenosis in these areas.
Pulmonary Artery Stenosis and Pulmonary Hypertension	Surgical interventions, catheterization, or hybrid procedures can be considered to manage pulmonary artery stenosis and associated pulmonary hypertension.
Abnormal Skin Healing	Careful postoperative evaluation of wounds is necessary due to potential delayed wound healing. Surgical stitches should be placed to avoid traction and should remain in place for a specified duration.
Hernias	Surgical repair using supporting mesh can minimize the recurrence of hernias.
Pectus Excavatum	Surgical intervention is indicated when pectus excavatum is severe and medically necessary.
Scoliosis	Orthopedic management, including surgery if needed, is recommended for scoliosis.
Pes Planus	Orthotics can be used to manage flat feet (pes planus).
Ocular Issues	Ophthalmologic management by specialists familiar with connective tissue disorders is essential. This includes aggressive refraction, visual correction, and evaluation for keratoconus.
Emphysema	Symptomatic treatment of emphysema is advised, and caution should be exercised with positive pressure ventilation due to the potential progression of emphysematous changes.
General Well-Being	Maintaining aerobic activity in moderation, such as swimming, is encouraged. Psychological issues, particularly in adolescence, may require psychological guidance.

Regular surveillance is crucial to monitoring the progression of ATS and its associated manifestations. Echocardiography is recommended every three months until the age of five years, followed by annual assessments if aortic diameters are within normal limits (for arterial tortuosity, aneurysms, stenosis, or dilatation of aortic root). MRA or CT scans with 3D reconstructions may be done annually from birth or the time of diagnosis and every three years in older children and adults under stable conditions. An echocardiogram should be performed as advised by a cardiologist for pulmonary hypertension. Blood pressure measurement should be conducted at each visit for systemic hypertension. Orthodontic evaluation is recommended during the eruption of permanent dentition for dental crowding. Radiographs should be taken during periods of rapid growth, such as the first two years of life and during puberty, to evaluate the progression of scoliosis. Routine follow-up with an ophthalmologist specializing in connective tissue disorders is necessary to monitor refractive errors and keratoconus. Baseline lung function should be assessed at the age of 18 years, with repeat exams based on symptomatology.

Certain agents and circumstances should be avoided to minimize risks: (1) Contact sports, competitive sports, and isometric exercise should be avoided. (2) Scuba diving should be avoided due to pressure differences and the need for positive pressure ventilation. (3) Agents that stimulate the cardiovascular system, including routine use of decongestants, should be avoided. (4) Tobacco use should be avoided due to increased cardiovascular and pulmonary risks and premature skin aging. (5) Sun tanning should be avoided to prevent premature skin aging.

It is important to evaluate the older and younger siblings of an individual with ATS to identify those who might benefit from surveillance and preventive measures. Molecular genetic testing can clarify the genetic status of at-risk siblings if the pathogenic variants in the family are known. If not, clinical evaluation and appropriate genetic testing can help determine their genetic status. Limited data are available on managing pregnancies in individuals with ATS. Preconception counseling should address potential risks to both the mother and fetus. Risks to the mother include aortic root dilatation and dissection, while medication-associated risks to the fetus need to be considered. Ideally, antihypertensive medications that are safe for pregnancy should be used, and elective aortic repair might be considered before conception if the aortic root diameter reaches a certain threshold. Increased surveillance and careful monitoring are advised during pregnancy and delivery.

Challenges and future directions

Despite the increasing knowledge and research efforts in the field of ATS, several limitations and areas for further exploration still exist. Currently, there are no specific targeted therapies available for ATS. The management approaches primarily involve symptom relief, blood pressure control, and surgical interventions. Advancements in understanding the molecular mechanisms driving ATS may lead to the development of novel therapeutics specifically targeting the underlying pathophysiology of the condition. Due to the rarity of ATS, conducting large-scale clinical trials to evaluate the efficacy of different treatment approaches poses a significant challenge. Collaborative efforts, the establishment of international registries, and the sharing of clinical data will be instrumental in overcoming this limitation and gathering comprehensive data for evidence-based management strategies.

The variable clinical presentation and progression of ATS necessitate an individualized approach to management. Each patient may require tailored interventions and treatment plans based on their specific arterial complications, associated symptoms, and overall health status. Implementing a multidisciplinary team approach involving cardiologists, geneticists, surgeons, and other specialists is crucial in providing optimal care. Long-term follow-up studies and natural history assessments are essential to evaluate the progression of arterial complications, identify potential predictors of adverse outcomes, and optimize management strategies. These studies can help guide the frequency of imaging surveillance, identify the appropriate timing of interventions, and improve prognostic evaluation. The impact of ATS on individuals and their families goes beyond the physical manifestations. Attention must be given to the psychosocial aspects, including emotional well-being, quality of life, and access to support networks and counseling services. Integrating psychological support within the management framework can have a significant positive impact on the overall care of ATS patients.

## Conclusions

ATS is a rare genetic disorder inherited in an autosomal recessive manner and affects both genders. It primarily presents in childhood, disrupting blood circulation, increasing shear stress, and leading to complications such as atherosclerosis and strokes. The genetics of ATS are closely linked to mutations in the SLC2A10 gene, which disrupt collagen and elastin synthesis, weakening the arteries and leading to upregulated TGFβ signaling. Arterial tortuosity, a complex phenomenon influenced by mechanical and anatomic factors, has been recognized as a hallmark of genetic arteriopathies, including ATS. Recent studies emphasize the potential of arterial tortuosity as a prognostic indicator for adverse cardiovascular events, particularly in conditions such as Marfan syndrome. While there are no specific clinical practice guidelines for ATS, strategies focus on addressing specific symptoms and risks associated with the condition. Regular surveillance and imaging play a crucial role in monitoring disease progression and identifying potential complications. However, the lack of targeted therapies remains a challenge, and further research into the molecular mechanisms of ATS could pave the way for novel treatment approaches.

## References

[1] Ertugrul A (1967). Diffuse tortuosity and lengthening of the arteries. Circulation.

[2] Palanca Arias D, Ayerza Casas A, Clavero Adell M (2022). Arterial tortuosity syndrome (variants in SLC2A10 gene) in two pediatric patients in the same city of Spain: a case report. Bull Natl Res Cent.

[3] Beyens A, Albuisson J, Boel A (2018). Arterial tortuosity syndrome: 40 new families and literature review. Genet Med.

[4] Naunheim MR, Walcott BP, Nahed BV, MacRae CA, Levinson JR, Ogilvy CS (2011). Arterial tortuosity syndrome with multiple intracranial aneurysms: a case report. Arch Neurol.

[5] Liang M, Wen H, Li S (2021). Two fetuses in one family of arterial tortuosity syndrome: prenatal ultrasound diagnosis. BMC Pregnancy Childbirth.

[6] Callewaert BL, Willaert A, Kerstjens-Frederikse WS (2008). Arterial tortuosity syndrome: clinical and molecular findings in 12 newly identified families. Hum Mutat.

[7] Coucke PJ, Willaert A, Wessels MW (2006). Mutations in the facilitative glucose transporter GLUT10 alter angiogenesis and cause arterial tortuosity syndrome. Nat Genet.

[8] Ritelli M, Chiarelli N, Dordoni C (2014). Arterial tortuosity syndrome: homozygosity for two novel and one recurrent SLC2A10 missense mutations in three families with severe cardiopulmonary complications in infancy and a literature review. BMC Med Genet.

[9] Del Corso L, Moruzzo D, Conte B (1998). Tortuosity, kinking, and coiling of the carotid artery: expression of atherosclerosis or aging?. Angiology.

[10] Morris SA (2015). Arterial tortuosity in genetic arteriopathies. Curr Opin Cardiol.

[11] Weibel J, Fields WS (1965). Tortuosity, coiling, and kinking of the internal carotid artery: II. Relationship of morphological variation to cerebrovascular insufficiency. Neurology.

[12] Han HC (2012). Twisted blood vessels: symptoms, etiology and biomechanical mechanisms. J Vasc Res.

[13] Loeys BL, Schwarze U, Holm T (2006). Aneurysm syndromes caused by mutations in the TGF-beta receptor. N Engl J Med.

[14] Ágg B, Szilveszter B, Daradics N (2020). Increased visceral arterial tortuosity in Marfan syndrome. Orphanet J Rare Dis.

[15] Morris SA, Orbach DB, Geva T, Singh MN, Gauvreau K, Lacro RV (2011). Increased vertebral artery tortuosity index is associated with adverse outcomes in children and young adults with connective tissue disorders. Circulation.

[16] Morris SA, Payne WA, Lacro RV (2014). Vertebral artery tortuosity in Turner syndrome: is tortuosity a component of the aortopathy phenotype?. J Cardiovasc Magn Reson.

[17] van de Laar IM, Oldenburg RA, Pals G (2011). Mutations in SMAD3 cause a syndromic form of aortic aneurysms and dissections with early-onset osteoarthritis. Nat Genet.

[18] Yasmeen S, Lund K, De Paepe A (2014). Occipital horn syndrome and classical Menkes Syndrome caused by deep intronic mutations, leading to the activation of ATP7A pseudo-exon. Eur J Hum Genet.

[19] Franken R, El Morabit A, de Waard V (2015). Increased aortic tortuosity indicates a more severe aortic phenotype in adults with Marfan syndrome. Int J Cardiol.

[20] Ciurică S, Lopez-Sublet M, Loeys BL (2019). Arterial tortuosity. Hypertension.

[21] Humphrey JD, Eberth JF, Dye WW, Gleason RL (2009). Fundamental role of axial stress in compensatory adaptations by arteries. J Biomech.

[22] Eleid MF, Guddeti RR, Tweet MS (2014). Coronary artery tortuosity in spontaneous coronary artery dissection: angiographic characteristics and clinical implications. Circ Cardiovasc Interv.

[23] Faiyaz-Ul-Haque M, Zaidi SH, Al-Sanna N (2009). A novel missense and a recurrent mutation in SLC2A10 gene of patients affected with arterial tortuosity syndrome. Atherosclerosis.

[24] Wessels MW, Catsman-Berrevoets CE, Mancini GM (2004). Three new families with arterial tortuosity syndrome. Am J Med Genet A.

[25] Bhat V (2014). Arterial tortuosity syndrome: an approach through imaging perspective. J Clin Imaging Sci.

[26] Bhat V, Muzrakchi AA (2008). Meandering vessels: a sign of arterial tortuosity on plain chest radiography. Heart Views.

[27] Lotmar W, Freiburghaus A, Bracher D (1979). Measurement of vessel tortuosity on fundus photographs. Albrecht Von Graefes Arch Klin Exp Ophthalmol.

[28] Bullitt E, Gerig G, Pizer SM, Lin W, Aylward SR (2003). Measuring tortuosity of the intracerebral vasculature from MRA images. IEEE Trans Med Imaging.

[29] Smedby O, Högman N, Nilsson S, Erikson U, Olsson AG, Walldius G (1993). Two-dimensional tortuosity of the superficial femoral artery in early atherosclerosis. J Vasc Res.

[30] Adriaans BP, Heuts S, Gerretsen S (2018). Aortic elongation part I: the normal aortic ageing process. Heart.

[31] Natoli AK, Medley TL, Ahimastos AA, Drew BG, Thearle DJ, Dilley RJ, Kingwell BA (2005). Sex steroids modulate human aortic smooth muscle cell matrix protein deposition and matrix metalloproteinase expression. Hypertension.

[32] Martins HF, Mayer A, Batista P, Soares F, Almeida V, Pedro AJ, Oliveira V (2018). Morphological changes of the internal carotid artery: prevalence and characteristics. A clinical and ultrasonographic study in a series of 19 804 patients over 25 years old. Eur J Neurol.

[33] Factor SM, Minase T, Cho S, Fein F, Capasso JM, Sonnenblick EH (1984). Coronary microvascular abnormalities in the hypertensive-diabetic rat. A primary cause of cardiomyopathy?. Am J Pathol.

[34] Li Y, Shen C, Ji Y, Feng Y, Ma G, Liu N (2011). Clinical implication of coronary tortuosity in patients with coronary artery disease. PLoS One.

[35] Li Y, Qadir Nawabi A, Feng Y, Ma G, Tong J, Shen C, Liu N (2018). Coronary tortuosity is associated with an elevated high-sensitivity C-reactive protein concentration and increased risk of ischemic stroke in hypertensive patients. J Int Med Res.

[36] Wang H-F, Wang D-M, Wang J-J (2017). Extracranial internal carotid artery tortuosity and body mass index. Front Neurol.

[37] Mochida M, Sakamoto H, Sawada Y (2006). Visceral fat obesity contributes to the tortuosity of the thoracic aorta on chest radiograph in poststroke Japanese patients. Angiology.

[38] Shang K, Chen X, Cheng C, Luo X, Xu S, Wang W, Liu C (2022). Arterial tortuosity and its correlation with white matter hyperintensities in acute ischemic stroke. Neural Plast.

[39] Soikkonen K, Wolf J, Mattila K (1995). Tortuosity of the lingual artery and coronary atherosclerosis. Br J Oral Maxillofac Surg.

[40] Hayes SN, Kim ES, Saw J (2018). Spontaneous coronary artery dissection: current state of the science: a scientific statement from the American Heart Association. Circulation.

[41] Liang JJ, Prasad M, Tweet MS (2014). A novel application of CT angiography to detect extracoronary vascular abnormalities in patients with spontaneous coronary artery dissection. J Cardiovasc Comput Tomogr.

[42] Olin JW (2017). Expanding clinical phenotype of fibromuscular dysplasia. Hypertension.

